# Positive childhood experiences reduce suicide risk in Japanese youth with ASD and ADHD traits: a population-based study

**DOI:** 10.3389/fpsyt.2025.1566098

**Published:** 2025-04-30

**Authors:** Masaki Adachi, Michio Takahashi, Hiroyuki Mori

**Affiliations:** ^1^ Department of Psychology, Meiji Gakuin University, Minao-Ku, Tokyo, Japan; ^2^ Research Department, Institute of Child Developmental Science Research, Hamamatsu, Shizuoka, Japan; ^3^ Department of Neuropsychiatry, Graduate School of Medicine, Hirosaki University, Hirosaki, Aomori, Japan; ^4^ Smart-Aging Research Center, Tohoku University, Sendai, Miyagi, Japan; ^5^ Faculty of Humanities, Saitama Gakuen University, Kawaguchi, Saitama, Japan

**Keywords:** positive childhood experiences, autism spectrum disorder, attention deficit/hyperactivity disorder, suicide-related behaviors, youth mental health

## Abstract

**Objective:**

This study investigated the combined influence of autism spectrum disorder (ASD) traits, attention-deficit/hyperactivity disorder (ADHD) traits, and positive childhood experiences (PCEs) on suicide-related behaviors in a large, representative sample of Japanese adolescents and young adults. Additionally, it explored the role of PCEs in mitigating the risks associated with neurodivergent traits.

**Methods:**

Data were collected from 5,000 individuals aged 16–25 years using validated scales to measure ASD traits, ADHD traits, PCEs, and suicide-related behaviors, including suicidal ideation and attempts. Hierarchical regression analysis was conducted in multiple steps to assess the influence of these variables. Interaction effects between PCEs and neurodivergent traits were examined to determine potential moderating effects.

**Results:**

ASD traits and ADHD traits were positively associated with suicidal ideation, with the highest risks observed among individuals with elevated levels of both traits. The inclusion of PCEs demonstrated a significant negative association with suicidal ideation, indicating that individuals with more PCEs reported lower levels of suicidal ideation. PCEs also reduced the strength of the associations of ASD traits (from β = 0.180 to β = 0.092) and ADHD traits (from β = 0.216 to β = 0.185) with suicidal ideation. Interaction analyses showed that the protective effect of PCEs on suicidal ideation was particularly pronounced among individuals with high levels of ADHD traits. Simple slope analyses demonstrated that higher levels of PCEs were significantly associated with reduced suicidal ideation for those with both low (β = −0.339, z = −18.61, *p* < 0.001) and high levels of ADHD traits (β = −0.475, z = −21.84, *p* < 0.001), with a stronger effect for the latter group.

**Conclusion:**

These findings highlight the cumulative and potentially compounding effects of ASD and ADHD traits on suicide risk while underscoring the critical protective role of PCEs. PCEs can mitigate emotional dysregulation and impulsivity, particularly in individuals with high levels of ADHD traits, thus reducing suicide-related behaviors. This study underscores the importance of fostering PCEs as part of targeted interventions to promote resilience and mental health in vulnerable populations.

## 1 Introduction

Suicidal behavior is a significant global health concern, particularly among adolescents and young adults (1). According to the World Health Organization (2), approximately 800,000 individuals die by suicide each year, making it the second leading cause of death among individuals aged 15–29 years. In Japan, suicide is the primary cause of death among individuals aged 15–34, a characteristic not observed in other G7 nations, which highlights the critical need for targeted interventions to address this issue (3).

Numerous studies have sought to identify potential risk factors for suicide (4). Among these, neurodevelopmental conditions such as autism spectrum disorder (ASD) and attention-deficit/hyperactivity disorder (ADHD) have been associated with an elevated risk of suicidal behavior (5, 6). ASD is characterized by impairments in social interaction and communication, restricted interests, and repetitive behaviors (7). Woolfenden et al. (8) found that adults with ASD have a mortality rate that is two to three times higher than that of the non-autistic population. A large-scale Canadian study reported that the mortality rate of individuals with ASD is approximately three times higher than that of their age-matched non-autistic counterparts, with adjusted relative risks of 3.13 (95% confidence interval [CI] 2.58–3.79) for males and 3.12 (95% CI 2.35–4.13) for females (9). Suicide has been identified as a significant contributor to this premature mortality (10).

Individuals with ASD are disproportionately prone to suicidal thoughts and behaviors, as clearly seen in the past decade (11). Furthermore, research suggests that a clinical diagnosis of ASD and elevated undiagnosed ASD traits within the general population are associated with greater susceptibility to suicidal ideation and attempts (11–14). Estimates of suicidality in children and adolescents with ASD vary widely (10.9%–50%) depending on the assessment tools used (15). Additionally, research indicates that 7%–15% of suicide cases in the general population involve individuals with an ASD diagnosis (16).

Cassidy et al. (14) demonstrated that ASD traits contribute to increased suicide risk through mechanisms including cognitive inflexibility, impaired problem-solving under stress, heightened feelings of entrapment, and increased vulnerability to social isolation, bullying, and abuse. Moreover, individuals with ASD traits frequently engage in camouflaging strategies to socially adapt, exacerbating psychological distress and significantly elevating suicide mortality approximately eleven times compared to the general population (14).

ADHD, the most prevalent neurodevelopmental condition, affects approximately 5.9% of youths and 2.5% of adults worldwide (17). It is diagnosed based on developmentally excessive and impairing levels of hyperactivity, inattention, and/or impulsivity (7). Like ASD, ADHD has been associated with an increased risk of premature death, including suicide (18). A large-scale population-based cohort study found that individuals with ADHD exhibit significantly higher rates of suicidal behaviors, with a nearly five-fold increased incidence compared to the general population, which substantially escalates in the presence of comorbid psychiatric conditions, highlighting the complex pathways underlying suicidality in ADHD populations (19). Proposed mechanisms underlying this heightened risk include impulsivity, emotional dysregulation, impaired decision-making abilities, social difficulties, educational underachievement, and significant psychosocial distress (17). Moreover, ADHD symptoms, even those below clinical diagnostic thresholds, significantly contribute to suicide-related behaviors (20, 21). Indeed, a recent meta-analysis (22) demonstrated that ADHD is the second mental disorder most strongly associated with suicidal ideation, surpassed only by depression (ADHD: d = 0.54 [95% CI, 0.34–0.75]; depression: d = 0.90 [95% CI, 0.71–1.09]).

Despite these significant findings, no study has examined the combined influence of these traits on suicidal ideation and behaviors. The prevalence of ADHD among individuals diagnosed with ASD varies considerably, with estimates ranging from 28.2% to 87% (23–26). However, all of these studies consistently state that a notable proportion of individuals with ASD also meet the diagnostic criteria for ADHD. Research has also shown that 15%–25% of children diagnosed with ADHD experience comorbid social difficulties associated with ASD (27, 28). The frequent comorbidity of ASD and ADHD suggests that these conditions may interact in ways that exacerbate the risk of adverse outcomes, including suicidal behavior. Kotte et al. (28) found that approximately 18.2% of children with ADHD exhibited ASD traits compared with only 0.87% of the control group. Importantly, children with ADHD who exhibit ASD traits demonstrated significantly greater impairments in psychological, social, educational, and cognitive domains than those who did not exhibit such traits, including higher rates of emotional dysregulation, more severe social difficulties, and academic underachievement. These findings suggest that even subclinical levels of ASD traits can substantially impact children with ADHD, compounding their overall level of impairment. Thus, it is important to evaluate ASD traits not only in clinical populations but also in subclinical or general populations to fully grasp their broader impact. Craig et al. (29) further demonstrated that individuals with both ASD and ADHD exhibit unique clinical profiles, including heightened emotional and behavioral problems and more severe adaptive behavior impairments compared with individuals with a single condition. This compounded severity in certain domains, such as social and daily living skills, highlights the potential for ASD and ADHD traits to interact synergistically, creating more adverse outcomes than either condition alone. Therefore, it is crucial to examine both conditions concurrently and scrutinize their compounded relationships rigorously.

Another important factor contributing to this compounded risk is the heightened susceptibility to experiencing Adverse Childhood Experiences (ACEs) among individuals with ASD and ADHD. Children with ADHD show significantly higher prevalence and severity of ACEs compared to peers without ADHD, with a clear graded relationship between the number of ACEs experienced and ADHD symptom severity (30). Similarly, children with ASD experience significantly more ACEs, particularly socioeconomic hardship, parental divorce, familial mental health issues, and bullying, compared to their neurotypical counterparts (31). Such ACEs are associated with increased risks for depression, anxiety, comorbid psychiatric conditions, and suicidality in these populations (31, 32). Additionally, both ASD and ADHD are associated with deficits in coping mechanisms, including impaired emotion regulation and limited adaptive responses to stress, further exacerbating vulnerability to negative outcomes following traumatic experiences (32).

While research on suicidality has predominantly focused on risk factors (4), protective factors have been underexplored (33, 34). Positive childhood experiences (PCEs) have recently been identified as a promising protective factor against various mental health challenges, including suicidality (35). PCEs, which were originally proposed solely as protective factors against ACEs, are increasingly recognized for their role in mitigating the negative effects of early-life adversity on developmental outcomes. Childhood trauma, including ACEs, has been shown to impair the prefrontal cortex (PFC) function, which is critical for executive processes such as self-regulation and attention control (36).

PCEs include supportive, nurturing experiences and relationships during childhood, which are believed to foster healthy development and positively influence mental health and relationship quality in adulthood. According to Bethell et al. (35), PCEs specifically include seven interpersonal and social experiences: (1) the ability to talk to family about feelings, (2) feeling supported by family during difficult times, (3) enjoying participation in community traditions, (4) feeling a sense of belonging in high school, (5) feeling supported by friends, (5) having at least two non-parent adults who showed genuine interest, and (6) feeling safe and protected by an adult at home. These seven PCE items were adapted from four validated subscales (psychological caregiving, educational engagement, cultural connectedness, and peer support) included in the Child and Youth Resilience Measure–28 (CYRM-28), which has demonstrated strong validity in culturally diverse contexts (35, 37, 38). Evidence suggests that PCEs reduce the risk of depression and promote social and emotional well-being throughout life (35). Children with diagnosed ADHD tend to have significantly higher ACEs than their peers and there is a correlation between ACE scores and the severity of ADHD symptoms (30). Studies from the 2011–2012 US National Survey of Children’s Health showed that autistic children also face more ACEs than their non-autistic peers, with 10.2% of autistic children experiencing four or more ACEs compared to 5.1% of their non-autistic peers (31). However, it remains unclear whether PCEs act as protective factors specifically within the relationship of neurodivergent traits with suicidality. Given the heightened risk of suicide-related behaviors associated with ASD and ADHD traits, as well as the potential mitigating role of PCEs, a comprehensive examination of these factors in the general population is critical.

Understanding how ASD and ADHD traits influence suicidality, both independently and in combination, can therefore inform early intervention strategies. Furthermore, exploring the protective capacity of PCEs in this context offers valuable insights into fostering resilience and reducing suicide risk. The primary aim of this study was therefore to examine the relationship between neurodivergent traits (specifically ASD and ADHD) and suicidality in adolescents and youth in the general population. As demonstrated, many studies have shown that ASD and ADHD traits have a significant impact on mental health (11, 20), extending even to the general population. Targeting the general population expands the possibility of early intervention and preventative support for undiagnosed individuals, who are at latent risk. Given the common comorbidity of ASD and ADHD symptoms, this study explored how the co-occurrence of these traits influences the risk of suicidality. In addition, this study will examine the interaction between risk factors (ASD and ADHD traits) and protective factors, particularly PCEs. By integrating both risk and protective factors, this study provides a more comprehensive understanding of the complex dynamics underlying suicide risk in people with high levels of ASD and ADHD traits and offers more personalized, effective interventions.

## 2 Materials and methods

### 2.1 Participants

In this study, 5,000 individuals aged 16–25 were recruited from panel data held by a major Japanese online research company. Participants were proportionally sampled according to the population distribution across Japan’s 47 prefectures, while ensuring approximately equal representation across each age group. The total number of male participants was 1,345, while 3,572 were female, and 83 did not respond. The mean age of the participants was 21.32 years. Prior to participation, individuals received online information detailing the purpose of the study and potential risks associated with participation, and informed consent was obtained from all participants. To ensure the quality of responses, an attention-check item was incorporated at the commencement of the survey. This item requested that respondents select the option ‘somewhat disagree’. Respondents who did not select the correct option were excluded from the subsequent analysis. The study’s objective was to examine the associations between ASD and ADHD traits and suicidality within the general population; therefore, no exclusion criteria based on psychiatric or neurodevelopmental diagnoses were applied. Comprehensive participant characteristics and regional distribution data are provided in **Supplementary Table 1**; **Table 1**.

**Table 1:** Demographic variables and descriptive statistics.

Variables	n (%)	Mean (SD)	Range
Age		21.3 (2.8)	16–25
16–20	1,837 (36.7)	―	―
21–25	3,163 (63.3)	―	―
Gender			
Male	1,345 (26.9)	―	―
Female	3,572 (71.4)	―	―
Non-binary	83 (1.6)	―	―
SES			
Upper class	472 (9.4)	―	―
Middle class	3,880 (77.6)	―	―
Lower class	648 (13.0)	―	―
Employment Status			
Employed	1,485 (28.2)	―	―
Student	2,522 (50.4)	―	―
Unemployed	316 (6.3)	―	―
Part-time employment	538 (10.8)	―	―
Homemaker or household helper	96 (1.9)	―	―
Other	43 (0.9)	―	―
ASD traits (AQ-10)	―	4.0 (2.2)	0–10
ADHD traits (ASRS)	―	9.5 (4.8)	0–24
Positive Childhood Experiences	―	4.4 (2.4)	0–7
Suicide-related behaviors (Suicidal Ideation Scale)	―	8.9 (3.0)	6–18
Prevalence of suicidal ideation			
Across lifetime (yes:1, no:0)	Yes: 1,696 (33.9)	0.34 (0.5)	0–1
Past month (yes:1, no:0)	Yes: 444 (8.9)	0.09 (0.3)	0–1

SES, Socioeconomic Status; ASD, Autism Spectrum Disorder; AQ-10, Autism Spectrum Quotient-10; ADHD, Attention-Deficit/Hyperactivity Disorder; ASRS, Adult ADHD Self-Report Scale; PCEs, Positive Childhood Experiences; SD, Standard Deviation.

### 2.2 Measures

#### 2.2.1 Demographics

The questionnaire included basic demographic information such as age, gender, socioeconomic status (SES), type of employment, and prefecture of residence (**Table 1**).

#### 2.2.2 Suicide-related behaviors

A comprehensive evaluation of suicidal behaviors was conducted using the Shortened Suicide Ideation Scale (39). Using this scale, the researchers assessed the following aspects on a scale of 1 to 3: suicidal ideation, the balance between the desires to live and to die, suicidal urges, duration of suicidal ideation, frequency of suicidal ideation, and suicide attempts. The potential range of scores was 6–18. A higher score indicated a higher risk of suicide. This scale has been validated for reliability and validity in Japan (39). Additionally, the researchers inquired about both the lifetime and one-month prevalence of suicidal ideation.

#### 2.2.3 ASD traits

The shortened version of the Autism-Spectrum Quotient (AQ; 40) called the AQ-10 (41) was employed as a measure of ASD traits. For each item, respondents indicated their level of agreement on a four-point scale (1 = not at all true, 4 = very true). If they selected the two options considered highly indicative of ASD traits, they were awarded one point, while those who selected the two other options were awarded zero points. The score range was 0–10, with a higher score indicating a greater degree of ASD traits. This scale has been demonstrated to possess good reliability (Cronbach’s α = 0.61) and high discriminant validity (diagnostic discrimination accuracy = 88%; 41). The cutoff point was set at seven points.

#### 2.2.4 ADHD traits

The Adult ADHD Self-Report Scale-v1.1 (ASRS) is one of the most commonly employed questionnaires for assessing ADHD symptoms (42). The scale comprises 18 items based on the diagnostic criteria for ADHD outlined in the *Diagnostic and Statistical Manual of Mental Disorders*, 4^th^ Edition, Text Revision (43). Symptom frequency was assessed on a five-point Likert scale, with responses ranging from 0 (never) to 4 (very often). The ASRS comprises two subscales for inattention and hyperactivity-impulsivity, each of which contains nine items (44–46). In the present study, the researchers employed a shortened version of the ASRS comprising six items (47). The internal consistency of the ASRS was found to be 0.63–0.72, while the test-retest reliability was observed to be 0.58–0.77 (46). The ASRS demonstrated a high consistency with clinical diagnoses, and the area under the receiver operating characteristic curve was 0.90, indicating a high degree of discriminant validity (46). The Japanese version of the ASRS has been demonstrated to be reliable and valid (48). The potential range of the scores was 0–24. A higher score was indicative of more pronounced ADHD traits. The cutoff point was set at 14 points (46).

#### 2.2.5 Positive childhood experiences

The seven items proposed by Bethell et al. (35) were employed for evaluating PCEs. The original instruction provided to respondents was simply: “how often or how much as a child they experienced the following” (35). This instruction does not specify a particular age range, childhood period, or developmental stage beyond the general indication “as a child.” Respondents were asked to report how often or how much as a child they: (1) felt able to talk to their family about feelings, (2) felt their family stood by them during difficult times; (3) enjoyed participating in community traditions, (4) felt a sense of belonging in high school (not including those who did not attend school or were home schooled), (5) felt supported by friends, (6) had at least two non-parent adults who took genuine interest in them, and (7) felt safe and protected by at least one adult at home (35). The respondents were required to indicate whether they had experienced each of the seven items by selecting either “yes” (awarded one point) or “no” (awarded zero points). The potential scoring range was 0–7. A higher score indicated a greater number of PCEs. The psychometric properties of this seven-item PCEs measure have been previously validated. Bethell et al. (35) reported good internal consistency (Cronbach’s α = 0.77) and confirmed a single-factor structure. Convergent validity was demonstrated by significant associations between higher cumulative PCE scores and improved adult mental health outcomes, including reduced likelihood of depression and poor mental health, as well as greater likelihood of consistently receiving social and emotional support (35, **Supplementary Materials**).

### 2.3 Statistical analysis

#### 2.3.1 Preliminary analysis

The samples were divided into four groups based on the cutoff scores of the AQ-10 and ASRS: the group with suspected ASD (sASD) scored above the AQ-10 cutoff, the group with suspected ADHD (sADHD) was those who scored above the ASRS cutoff, the group with suspected ASD and ADHD (sASD+ADHD) scored above the cutoff for both the AQ-10 and the ASRS, and the group without suspected ASD or ADHD (non-sASD+ADHD) scored below the cutoff for both the AQ-10 and the ASRS. We did not collect data regarding formal clinical diagnoses of ASD or ADHD among participants; therefore, the classification into these groups was based solely on scores from the screening tools (AQ-10 and ASRS). Differences in the lifetime prevalence of suicidal ideation, past-month suicidal ideation, PCE scores, and suicidal ideation scale scores between these groups were examined using analysis of covariance (ANCOVA) controlling for age, SES, and gender. SES was assigned as a dummy variable with the following values: upper class = 1, middle class = 2, and lower class = 3, with a higher SES score indicating a higher level of economic hardship.

#### 2.3.2 Main analysis

The researchers conducted hierarchical multiple regression analyses using the suicidal ideation scale as the dependent variable. In step one, the control variables—gender, age, and SES—were entered into the model. In step two, ASD traits were added, followed by ADHD traits in step three and PCEs in step four. In step five, interaction terms were introduced to examine the potential interactions between ASD traits and PCEs (ASD × PCEs) and ADHD traits and PCEs (ADHD × PCEs). To address the potential multicollinearity arising from the inclusion of interaction terms, all explanatory variables were centered. Participants who did not specify their gender (n = 83) were excluded from the hierarchical regression analyses, as gender was included as a covariate in all regression models. Consequently, the final analytical sample consisted of 4,917 participants.

To clarify the nature of these interaction effects, simple slope analyses were conducted. This method permitted an examination of how the relationships between ASD/ADHD traits, PCEs, and suicidal ideation differed across the moderating variables (e.g., ± 1 standard deviation [SD] from the mean). Such analyses would identify whether and how the protective effects of PCEs extend to individuals with pronounced ASD and ADHD traits. This approach was particularly valuable for understanding whether PCEs exert consistent protective effects across all levels of neurodivergent traits or whether their influence varies for specific subgroups. Although previous research has established the general protective role of PCEs, their efficacy in populations with high levels of neurodivergent traits remains unclear. By focusing on these interactions, a simple slope analysis can offer critical insights into how PCEs and neurodivergent traits collectively shape suicidality under different conditions.

### 2.4 Ethical considerations

This study was conducted in accordance with the ethical guidelines set forth by the Ethics Committee of Meiji Gakuin University, which approved the research protocol (approval number: 20230024). Prior to their involvement in the study, all participants gave their informed consent. The participants were provided with comprehensive information regarding the study’s objectives, methodology, and the potential risks associated with the discussion of sensitive topics (i.e., suicidal ideation and behaviors). The participants were explicitly reminded of these potential risks before providing their responses. They were also informed of their right to withdraw at any time without negative consequences. All data were anonymized to protect participant confidentiality, and no personally identifiable information was collected. Despite the minimal risk posed by the study, the participants were informed that they could skip any questions they found distressing or discontinue their participation if necessary. The collected data were securely stored and used solely for academic research purposes.

## 3 Results

### 3.1 Preliminary analysis

The results of the preliminary analysis are summarized in **Table 2**. A comparative analysis was conducted to investigate differences in PCEs and suicidality across four groups: non-sASD+ADHD (n = 3,630), sASD (n = 481), sADHD (n = 582), and sASD+ADHD (n = 307), controlling for age, SES, and gender. The results of the *post hoc* comparisons using the Holm method are provided in **Supplementary Table 2**. The results of the preliminary analysis indicated that the sASD+ADHD group demonstrated the highest risks across all suicide-related indicators and the fewest PCEs (**Supplementary Figure 1**).

**Table 2:** Analysis of covariance results for PCEs, suicide-related behaviors across neurodivergent traits groups.

	non-sASD+ADHD (n =3630)	sASD (n = 481)	sADHD (n = 582)	sASD + ADHD (n = 307)	*F*	*p*	*η_p_^2^*	Multiple comparisons (Holm method)
	Mean (95%CI)							
PCEs	4.6 (4.6–4.7)	3.7 (3.5–3.9)	4.1 (3.9–4.3)	3.1 (2.9–3.4)	65.9	<.001	0.038	No suspicion > sADHD > sASD > sASD & ADHD
Suicide-related behaviors (Suicidal Ideation Scale)	8.5 (8.3–8.6)	9.6 (9.4–9.9)	9.9 (9.7–10.1)	11.1 (10.7–11.4)	119.2	<.001	0.067	sASD & ADHD > sASD, sADHD > No suspicion
Prevalence of Suicidal Ideation								
Across Lifetime (yes:1, no:0)	0.30 (0.29–0.32)	0.39 (0.34–0.42)	0.42 (0.39–0.46)	0.56 (0.50–0.60)	37.1	<.001	0.022	sASD & ADHD > sASD, sADHD > No suspicion
Past month (yes:1, no:0)	0.06 (0.06–0.07)	0.11 (0.09–0.14)	0.16 (0.14–0.18)	0.21 (0.18–0.24)	42.2	<.001	0.025	sASD & ADHD > sADHD > sASD > No suspicion

PCEs, Positive Childhood Experiences; ASD, Autism Spectrum Disorder; ADHD, Attention-Deficit/Hyperactivity Disorder; sASD, suspected ASD; sADHD, suspected ADHD; CI, Confidence Interval; ηp², Partial eta squared.

#### 3.1.1 PCEs

The model was significant, *F*(6, 4993) = 112.07, *p* < 0.001, with an adjusted *R*² of 0.118, indicating that 11.8% of the variance in PCE scores was due to the covariates and group membership. The main effect of group membership was significant. Age (*F*(1, 4993) = 12.05, *p* < 0.001) and SES (*F*(1, 4993) = 378.50, *p* < 0.001) were significant covariates, whereas gender was not (*F*(1, 4993) = 2.34, *p* = 0.125).

#### 3.1.2 Suicidal ideation scale

The overall model was significant, *F*(6, 4993) = 118.48, *p* < 0.001, with an adjusted *R*² of 0.125, indicating that the model explained 12.5% of the variance in the suicidal ideation scores. The main effect of group membership was significant. Age (*F*(1, 4993) = 11.40, *p* = 0.001), SES (*F*(1, 4993) = 238.35, *p* < 0.001), and gender (*F*(1, 4993) = 59.51, *p* < 0.001) were significant covariates.

#### 3.1.3 Lifetime prevalence of suicidal ideation

The overall model was significant, *F*(6, 4993) = 41.72, *p* < 0.001, with an adjusted *R*² of 0.047, indicating that the model explained 4.7% of the variance in the lifetime prevalence of suicidal ideation. The main effect of group membership was significant. SES (*F*(1, 4993) = 68.59, *p* < 0.001) and gender (*F*(1, 4993) = 55.83, *p* < 0.001) were significant covariates, but age was not (*F*(1, 4993) = 0.61, *p* = 0.436).

#### 3.1.4 Past-month suicidal ideation

The overall model was significant, *F*(6, 4993) = 33.52, *p* < 0.001, with an adjusted *R*² of 0.038, indicating that the model explained 3.8% of the variance in past-month suicidal ideation. The main effect of group membership was significant. SES (*F*(1, 4993) = 46.28, *p* < 0.001) and gender (*F*(1, 4993) = 14.34, *p* < 0.001) were significant covariates, but age was not (*F*(1, 4993) = 1.06, *p* = 0.304).

### 3.2 Main analysis

The results of the main analysis are presented in **Table 3**. The researchers performed a hierarchical regression analysis in multiple steps to identify the predictors of comprehensive suicide risk (suicidal ideation scale). In each step, different sets of variables were progressively added to assess their contributions. In the preliminary analysis, similar trends were observed in the relationships between the suicidal ideation scale results and the lifetime prevalence of suicidal ideation, past-month suicidal ideation, and ASD and ADHD traits. Therefore, the main analysis focused on the suicidal ideation scale as the outcome variable to ensure clarity and specificity in examining the relationships.

**Table 3:** Impact of neurodivergent traits and positive childhood experiences on suicidality.

	Step 1	Step 2	Step 3	Step 4	Step 5			
Variables	*β*	*β*	*β*	*β*	*β*	95% CI		VIF
						Lower class	Upper class	
Gender (0: male, 1: female)	0.097**	0.121**	0.111**	0.097**	0.095**	0.072	0.119	1.02
Age	−0.054**	−0.056**	−0.031*	−0.051**	−0.054**	−0.078	−0.031	1.05
Socioeconomic status	0.231**	0.201**	0.190**	0.092**	0.091**	0.062	0.12	1.11
ASD traits (AQ-10)	―	0.260**	0.180**	0.092**	0.090**	0.064	0.117	1.26
ADHD traits (ASRS)	―	―	0.216**	0.185**	0.181**	0.154	0.208	1.27
Positive Childhood Experiences	―	―	―	−0.400**	−0.407**	−0.437	−0.378	1.30
ASD traits × PCEs	―	―	―	―	0.020	−0.007	0.048	1.20
ADHD traits × PCEs	―	―	―	―	−0.073**	−0.101	−0.045	1.22
Δ*R* ^2^	―	0.066**	0.039**	0.136**	0.004**			
*R* ^2^	0.062**	0.128**	0.167**	0.304**	0.308**			

^**^ *p* < 0.01, ^*^ *p* < 0.05.

ASD, Autism Spectrum Disorder; AQ-10, Autism Spectrum Quotient-10; ADHD, Attention-Deficit/Hyperactivity Disorder; ASRS, Adult ADHD Self-Report Scale; PCEs, Positive Childhood Experiences; CI, Confidence Interval; VIF, Variance Inflation Factor.

In step one, gender (coded as 0 = male, 1 = female), age, and SES were entered as control variables. Female gender (with male as the reference category) was positively associated with suicidal ideation. positively associated with suicidal ideation (*p* < 0.01), whereas age showed a negative association (*p* < 0.01), indicating that older participants tended to report lower levels of suicidal ideation. A higher SES score (i.e., greater economic hardship) was also associated with increased levels of suicidal ideation (*p* < 0.01). This model accounted for 6.2% of the variance in suicidal ideation (*p* < 0.01).

ASD traits were added in step two, demonstrating a significant increase in the explained variance (6.6%, *p* < 0.01). ASD traits were positively associated with suicidal ideation (*p* < 0.01), suggesting that individuals with higher levels of ASD traits reported greater suicidal ideation scores. The cumulative variance explained by the model at this stage was 12.8% (*p* < 0.01). In step three, ADHD traits were introduced, leading to a further increase in the explained variance (3.9%, *p* < 0.01). The analysis showed a positive association between ADHD traits and suicidal ideation (*p* < 0.01). At this point, the model explained 16.7% of the variance in suicidal ideation (*p* < 0.01).

The addition of PCEs in step four showed their significant negative association with suicidal ideation scores (*p* < 0.01). This indicated that individuals who reported more positive experiences during childhood tended to have lower suicidal ideation scores. It also led to a reduction in the strength of the association between both ASD traits (from β = 0.18 to β = 0.092) and ADHD traits (from β = 0.216 to β = 0.185) with suicidal ideation. Including PCEs increased the explained variance by 13.6% (*p* < 0.01), bringing the total variance explained to 30.4% (*p* < 0.01).

To explore potential moderating effects, the interaction terms between ASD/ADHD traits and PCEs were introduced in step five. This explained an additional 0.4% of the variance (*p* < 0.01). The interaction between ADHD traits and PCEs in particular suggested that the protective influence of PCEs on suicidal ideation varies depending on individuals’ level of ADHD traits. This final model explained 30.8% of the variance in suicidal ideation (*p* < 0.01). Furthermore, each step showed a statistically significant improvement in the model’s fit, as evidenced by increases in *R^2^*. Throughout the analyses, the variance inflation factor values were consistently below a level of two, indicating no substantial concerns about multicollinearity.

**Figure 1:**
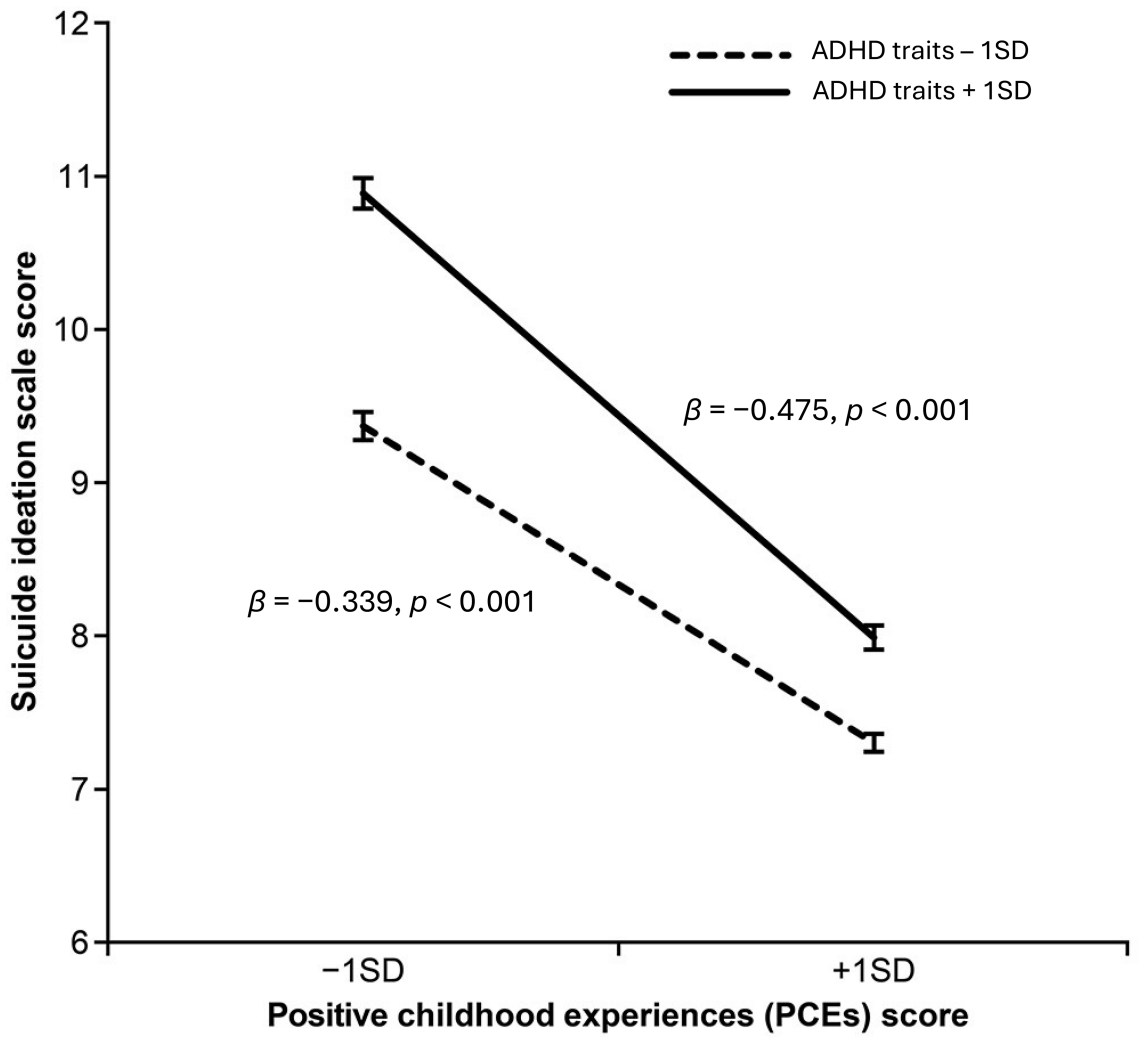
Interaction Effect of ADHD Traits and Positive Childhood Experiences (PCEs) on Suicide Ideation Scores. The solid line represents individuals with high levels of ADHD traits (+1 SD), whereas the dashed line represents individuals with low levels of ADHD traits (−1 SD). Higher PCE scores were associated with lower suicide ideation scores across both groups, with a stronger protective effect observed in individuals with high levels of ADHD traits. Error bars indicate standard errors. ADHD, Attention-Deficit/Hyperactivity Disorder; PCEs, Positive Childhood Experiences; SD, Standard Deviation.

To further investigate the significant effects of the interactions between ADHD traits and PCEs, simple slope analyses were conducted, stratified by ADHD traits at ±1 SD from the mean. The analyses revealed distinct patterns in the relationship between PCEs and suicide-related behaviors across different levels of ADHD trait occurrence (**Figure 1**). For participants with low levels of ADHD traits (−1 SD), PCEs demonstrated a significant protective effect against suicidality (b = −0.433, β = −0.339, *se* = 0.023, *z* = −18.61, *p* < 0.001), indicating that higher levels of PCEs are associated with lower levels of suicide-related behaviors. For participants with high levels of ADHD traits (+1 SD), PCEs also exhibited a significant protective effect (b = −0.608, β = −0.475, *se* = 0.028, *z* = −21.84, *p* < 0.001), demonstrating a greater impact compared to those with low ADHD levels. The interaction between ADHD traits and PCEs demonstrates that the protective influence of PCEs on suicidal ideation varies depending on the level of ADHD traits present.

## 4 Discussion

To our knowledge, this study is the first to examine the combined influence of ASD and ADHD traits on the risk of suicide-related behaviors by simultaneously analyzing both sets of traits in a large, representative sample of adolescents and young adults. While prior research has explored the individual impact of ASD and ADHD traits on suicidality (5, 12), examining these traits together offers unique insights into how their overlapping effects may heighten the risk of suicidal ideation and behaviors. This approach allows for a more comprehensive understanding of the cumulative and potentially compounding effects of ASD and ADHD traits, highlighting the importance of considering both conditions when assessing suicide risk among individuals who exhibit high levels of neurodivergent traits, even when those traits are below clinical thresholds for diagnosis.

Additionally, this study was the first to demonstrate the protective role of PCEs within the context of ASD and ADHD traits and suicidality. Importantly, the results reveal an interaction between ADHD traits and PCEs, in which PCEs have a particularly significant protective effect for individuals with higher levels of ADHD traits. This finding suggests that PCEs are especially crucial for reducing suicide risk among adolescents and young adults who exhibit elevated ADHD traits, underscoring the need for supportive early-life experiences for mitigating suicidality within this population.

### 4.1 Group comparisons and suicidality

The preliminary group comparisons using ANCOVA added further nuance to the findings by demonstrating significant differences in suicidality levels and PCE scores among the examined groups. Participants with both ASD and ADHD traits (sASD+ADHD) reported the highest levels of suicidality and the lowest number of PCEs, compared to those with sASD, sADHD, or neither. This suggests that the combination of struggles with socializing (ASD) and inattentiveness or impulsivity (ADHD) contributes to a higher risk of suicidality. Recent evidence supports this interpretation. Sun et al. (49) reported that young adults with combined ASD and ADHD traits had significantly higher risks of suicidal thoughts and behaviors than those with either trait alone, emphasizing the compounding effects of co-occurring neurodevelopmental difficulties. Furthermore, Craig et al. (29) highlighted compounded difficulties in social interaction, emotional regulation, and executive functioning among individuals with combined ASD and ADHD traits, factors which likely exacerbate psychological distress and suicidal ideation.

Moreover, Hartley et al. (50) demonstrated through a meta-analysis that autistic individuals experience adverse childhood experiences (ACEs) at more than twice the rate of their non-autistic peers. Given that increased exposure to ACEs typically reduces the likelihood of accumulating protective, positive childhood experiences (PCEs), this finding provides critical context for our results. Youth with combined ASD and ADHD traits reported the fewest PCEs in our study, likely contributing further to their heightened suicidality. These integrated findings underscore the importance of tailored interventions that specifically address both the compounded vulnerabilities arising from ASD and ADHD traits and the critical need to enhance PCEs as protective factors against suicidality.

### 4.2 Neurodevelopmental conditions and suicidality

Even after accounting for SES, gender, and age, ASD traits were positively associated with higher scores on the suicide ideation scale. This finding aligns with previous research indicating that suicide risk among individuals with ASD is significantly higher than in the general population (10). Recent studies further clarify psychological mechanisms underlying this increased risk, particularly emphasizing executive functioning deficits, cognitive inflexibility, and social isolation. Cook et al. (51) demonstrated that executive functioning impairments, such as difficulties in planning, decision-making, and cognitive flexibility, independently predict suicidal ideation among transition-aged autistic youth, directly supporting our findings regarding the role of cognitive challenges in suicidality. Cassidy et al. (52) further complement this perspective by showing that camouflaging autistic traits indirectly increases suicidal thoughts through enhanced feelings of defeat and entrapment, reflecting the complex interplay between cognitive rigidity, psychological distress, and social challenges frequently experienced by individuals with ASD traits.

ADHD traits, especially impulsivity and emotional dysregulation, were significantly associated with suicidality, consistent with prior studies emphasizing these traits as critical factors in suicidality among individuals with ADHD (17, 20). Recent findings further clarify this relationship, highlighting attentional impulsiveness and the severity of inattentive symptoms as significant predictors of severe suicidal ideation among adults with ADHD (53). Specifically, attentional impulsiveness—difficulties focusing attention and controlling intrusive thoughts—may influence the escalation from suicidal ideation to more severe suicidal behaviors. Although our study did not directly examine the transition from ideation to behavior, the strong association observed between ADHD traits and suicidal ideation underscores impulsivity’s potential role as a critical mechanism in this pathway. The highest risk of suicide-related behaviors was observed among individuals exhibiting both ASD and ADHD traits, clearly indicating that the interplay between these conditions substantially heightens suicidality risk, aligning with prior clinical observations from ASD populations (23).

These recent findings highlight the importance of targeting both common and distinct psychological mechanisms—including cognitive inflexibility, emotional dysregulation, and impulsivity—to effectively mitigate suicidality risk in individuals with combined ASD and ADHD traits. By elucidating these psychological pathways, our findings support a more tailored, nuanced approach to interventions and preventive efforts within these vulnerable groups.

### 4.3 Interplay between ASD and ADHD traits

Further exploration of the interaction between ASD and ADHD traits revealed critical insights. The results of step three, in which ADHD traits were introduced into the model, demonstrated a noticeable reduction in the effect size of ASD traits on suicidal ideation (from β = 0.26 to β = 0.18, representing a 30.77% reduction). This substantial reduction surpasses the threshold (20%) suggested by Hosmer et al. (54) as indicating meaningful confounding or moderation effects. This statistical pattern aligns with previous studies demonstrating substantial symptom overlap and comorbidity between ASD and ADHD (29, 55). Craig et al. (29) highlighted that ASD and ADHD traits often overlap significantly, with the ASD+ADHD group exhibiting characteristics of both conditions, including more severe difficulties in adaptive behaviors and elevated hyperactive and impulsive behaviors. Similarly, Canals et al. (55) reported that approximately 32.8% of autistic children and 31.4% of those with subthreshold ASD exhibited comorbid ADHD traits, reinforcing the notion of substantial shared variance between these neurodevelopmental traits.

However, beyond shared symptomatology, the observed pattern suggests a distinct difference in how ADHD and ASD traits influence suicidal ideation. ADHD traits, characterized by impulsivity and emotional dysregulation, likely exert a direct influence on suicidal ideation (17, 20). Conversely, the effect of ASD traits might be more indirect. For instance, Cassidy et al. (52) demonstrated that autistic traits increase suicide risk through indirect pathways involving camouflaging behaviors, which intensify feelings of defeat and entrapment—both established psychological precursors to suicidal ideation. Thus, the reduced effect of ASD traits observed upon inclusion of ADHD traits in our analysis may indicate that ASD traits primarily contribute indirectly, potentially mediated through psychological mechanisms exacerbated by comorbid ADHD traits and associated emotional and cognitive difficulties.

These nuanced findings underscore the importance of distinguishing between direct (ADHD) and indirect (ASD) pathways when interpreting their combined impact on suicidality. The high comorbidity between ASD and ADHD can complicate the diagnostic process and clinical interventions, necessitating tailored strategies that address both their overlapping and unique mechanisms of influence. Future research should focus on disentangling the direct and indirect contributions of ASD and ADHD traits, as well as their shared mechanisms, to better inform clinical practice. Additionally, studies should further explore how ADHD traits mediate or moderate the critical relationship between ASD traits and developmental or mental health outcomes. Such interactions must be better understood to design effective interventions and support systems. Individuals with high levels of both ASD and ADHD traits represent a particularly vulnerable population that requires targeted strategies to mitigate risks and promote resilience. Clarifying these underlying psychological pathways can significantly enhance targeted prevention and intervention strategies for these vulnerable populations.

### 4.4 Protective role of PCEs

The above findings suggest that PCEs exert a protective effect against suicidality in both diagnosed individuals and those in the general population who exhibit pronounced levels of ASD and ADHD traits. These results align with previous research indicating that PCEs mitigate the impact of ACEs and promote both emotional resilience and emotion regulation, thereby supporting positive mental health outcomes (35). PCEs may be particularly beneficial for individuals at heightened risk due to their neurodivergent traits because such experiences improve emotion regulation and reduce impulsivity, both of which are key factors in addressing suicidality (56). Furthermore, clinical studies have demonstrated that PCEs such as supportive relationships and community involvement positively influence emotional regulation in individuals with ADHD traits (57). Specifically, Lowe et al. (57) discovered that PCEs are indirectly associated with improved emotional regulation in adults with ADHD through the enhanced social support they promote. Higher PCE scores were linked to increased support for tangible needs, self-esteem, and belonging, all of which are vital for improving emotional regulation. For instance, tangible support significantly reduced emotional dysregulation (β = −0.5, 95% CI [−1.07, −0.02]), and support for self-esteem (β = −0.61, 95% CI [−1.08, −0.27]) and belonging (β = −0.43, 95% CI [−0.87, −0.05]) also made substantial contributions. These findings highlight the long-term benefits of PCEs in fostering resilience and enabling the development of effective emotional regulation strategies, which are particularly crucial for individuals with ADHD traits.

Based on these results, the mechanisms identified by Lowe et al. (57) in ADHD populations may also be applicable to individuals with ASD traits. Specifically, the findings of this study have suggested that PCEs can address the critical factors underlying suicidality by reducing impulsivity and improving emotional regulation through enhanced social support. As previously mentioned, the social isolation and communication difficulties that are common in ASD likely contribute to feelings of despair, increasing the vulnerability of individuals with ASD to suicidality (13). The effects of PCEs may mitigate such feelings. Notably, no prior research has explicitly examined the relationship between ASD and PCEs. This study was among the first to suggest that PCEs may also play a protective role in individuals with pronounced ASD traits. Understanding and leveraging these protective effects is an important factor in developing targeted interventions aimed at reducing risk and promoting resilience in these vulnerable populations.

However, the ANCOVA results of this study further revealed that individuals with both ASD and ADHD traits (sASD+ADHD) reported significantly fewer PCEs than those with either trait alone or with neither condition. This lack of protective experiences may explain the elevated risk of suicidality in this group. Given the significant role of PCEs in bolstering mental health, these findings underscore the critical need for early interventions that foster supportive and nurturing environments for young individuals with ASD and ADHD traits.

### 4.5 Interaction effects

Across both ADHD trait levels, PCEs showed a robust and statistically significant protective effect on suicide-related behaviors, with a stronger effect observed among participants with higher levels of ADHD traits (β = −0.475) compared with those with lower levels (β = −0.339). Although the difference between these groups (Δβ = 0.136) represents a small difference in effect size according to conventional standards (58), recent meta-analytic evidence underscores that even modest effects can carry significant clinical importance when addressing severe outcomes such as suicidal ideation (59). Franklin and colleagues explicitly highlight that, due to the serious implications associated with suicide-related outcomes, small protective influences should not be overlooked, particularly in vulnerable populations. The interaction between ADHD traits and PCEs suggests that the protective influence of PCEs against suicidal ideation varies depending on the level of ADHD traits.

As noted previously, ACEs may cause trauma in children. These neurobiological changes may result in ADHD-like symptoms even in individuals without a formal diagnosis (30). Cassiers et al. (36) discussed evidence showing that all forms of abuse—physical, emotional, and sexual—are associated with disruptions in brain development, particularly in the prefrontal cortex (PFC), which may lead to cognitive and behavioral symptoms resembling ADHD. These findings highlight the overlap between trauma-related symptoms and ADHD, complicating the accuracy of diagnosis. Recent concerns about the potential overdiagnosis of ADHD in certain populations further underscore the significance of this issue. Evidence suggests that a substantial proportion of potential ADHD overdiagnoses may result from the misidentification of trauma-related symptoms as ADHD symptoms (30). Both conditions share overlapping features, such as inattention, impulsivity, and emotional dysregulation (60). This diagnostic ambiguity is particularly relevant for individuals exposed to high levels of ACEs, who may exhibit ADHD-like symptoms driven primarily by trauma-induced PFC dysfunction rather than by a neurodevelopmental disorder.

The enhanced protective effect of PCEs observed in individuals with higher levels of ADHD traits may reflect the neuroplastic capacity of the PFC to recover or adapt when supported by positive environmental influences, as suggested by Segovia et al. (61). Moreover, this finding aligns closely with developmental theories such as the Differential Susceptibility and Vantage Sensitivity models (62), which propose that individuals with developmental vulnerabilities—such as elevated ADHD traits—are particularly sensitive to both adverse and supportive environmental influences, thereby benefiting disproportionately from positive childhood experiences. PCEs may mitigate the effects of trauma-induced dysfunction in the PFC, thereby alleviating the ADHD traits exacerbated by trauma exposure. For individuals with higher levels of ADHD traits, this neuroplasticity could explain why PCEs exert a stronger protective effect, as the buffering impact of these positive experiences may counteract the compounded challenges posed by trauma symptoms and ADHD traits. Thus, it is crucial to consider trauma exposure when evaluating ADHD symptoms. The present findings highlight the potential of targeted interventions aimed at enhancing PCEs to improve outcomes for individuals with high levels of ADHD traits. Future studies should further investigate the mechanisms by which PCEs influence neurodevelopment and their differential impact on ADHD traits in the presence of trauma-related experiences, as well as explore the practical implications of integrating these theoretical frameworks into clinical interventions.

### 4.6 Limitations and future directions

While this study offers significant insights into the relationship between neurodivergent traits, PCEs, and suicidality, several limitations warrant consideration.

#### 4.6.1 Lack of consideration of depression, comorbid conditions, and adverse childhood experiences

This study demonstrated that PCEs had a protective effect against suicidal behaviors across all levels of ASD and ADHD traits. Furthermore, an interaction effect indicated that this protective influence of PCEs was even more pronounced among individuals with higher levels of ADHD traits compared to those with lower levels. However, it did not explore the potential moderating effects of comorbid psychiatric conditions (e.g., anxiety, depression) frequently associated with neurodivergent traits. Recent research underscores the importance of considering depression, in particular, as a critical factor in understanding suicidality among individuals with ASD and ADHD traits. Gagliano et al. (63) emphasized that depressive disorders often remain undiagnosed or underdiagnosed in neurodevelopmental populations despite their significant role as mediators linking neurodevelopmental conditions with suicidal ideation and behavior. Depression frequently co-occurs with emotional dysregulation and cognitive deficits, exacerbating suicide risk among individuals with ASD and ADHD.

Furthermore, it is important to recognize that neurodevelopmental conditions share several underlying risk factors for suicidality, such as emotional dysregulation and impaired executive functions; however, each condition also has unique, condition-specific risk pathways. For instance, social isolation and impaired emotion recognition may particularly heighten suicide risk in individuals with ASD (11), while impulsivity and difficulties in inhibitory control significantly contribute to suicide risk among individuals with ADHD traits (21). Explicitly distinguishing these shared and distinct mechanisms in future studies would enhance interpretation of interaction effects and provide a more nuanced understanding of how comorbid conditions influence suicidality.

Another notable limitation is that this study emphasized PCEs as a primary protective factor but did not concurrently account for the potential impact of ACEs. Given that PCEs are generally negatively correlated with ACEs (35), it is plausible—although not certain—that individuals reporting higher levels of PCEs might have experienced fewer ACEs. Because we did not directly measure ACEs in this study, we cannot definitively conclude whether the observed protective association between PCEs and suicidal ideation was independently driven by PCEs or was partially attributable to unmeasured ACEs. Furthermore, we cannot rule out the possibility that our study participants represented a population with relatively low exposure to ACEs and relatively high PCEs. Future research should therefore simultaneously assess the effects of both PCEs and ACEs, enabling a more accurate evaluation of their independent and interactive contributions to suicidal ideation.

Thus, subsequent studies should comprehensively examine common and distinct mechanisms involving comorbid psychiatric conditions and ACEs to better clarify their complex roles in suicide-related behaviors among neurodivergent populations.

#### 4.6.2 Self-reported measures

The reliance on self-reported measures is a limitation of the present study. Self-report scales are inherently subjective and susceptible to recall biases, cognitive biases, and social desirability effects. This limitation is particularly salient among individuals with autistic traits, who often demonstrate atypical responses to emotional stimuli due to challenges in emotion recognition and regulation (64, 65). Autistic individuals may face specific difficulties in recognizing, interpreting, and describing their emotional experiences due to disruptions in emotion identification, appraisal, and monitoring processes (65). Such difficulties potentially reduce the reliability and accuracy of retrospective self-reported assessments of childhood experiences, including PCEs. The absence of an observed interaction between ASD traits and PCEs in the current study, contrasted with the observed interaction between ADHD traits and PCEs, might be partially explained by these emotion recognition and reporting difficulties specific to ASD traits. Therefore, future research could benefit from incorporating objective physiological measures or multi-method approaches (e.g., multi-informant assessments, physiological indicators) to enhance the accuracy and robustness of measurements.

#### 4.6.3 Cross-sectional design

This study employed a cross-sectional design, which precluded the ability to infer causality. Although the findings revealed significant associations between ASD/ADHD traits, PCEs, and suicidal ideation, the temporal sequence of these relationships remains unclear. Longitudinal studies are required to establish the potential causal pathways and examine how these relationships evolve, particularly during critical developmental periods.

#### 4.6.4 Lack of objective biological data

This study did not include objective biological or neurological measures that could provide additional insights into the mechanisms underlying the observed associations. Neuroimaging studies could further elucidate the role of PFC dysfunction in mediating the relationship between neurodivergent traits, trauma, and suicidality.

#### 4.6.5 Limited generalizability

The sample of participants was drawn from a population-based panel in Japan, which may limit the generalizability of the findings to other cultural or demographic contexts. Cross-cultural studies are therefore necessary to determine whether the observed relationships between these factors remain consistent across more diverse populations with varying socio-cultural norms and mental health resources.

#### 4.6.6 Limited scope of protective factors

Although this study focused on PCEs as a protective factor, other contextual and relational variables such as family cohesion, peer support, and access to mental health services may also play a critical role in mitigating the risk of suicidality. Expanding the range of potential protective factors under examination could yield further insights into appropriate intervention strategies.

### 4.7 Future directions

Building upon these limitations, future research should employ longitudinal designs to explore the causal relationships and developmental trajectories between neurodivergent traits, PCEs, and suicidality. It should also integrate biological measures such as neuroimaging or biomarkers to investigate the neurobiological mechanisms underlying these relationships. Moreover, future research should explicitly measure depression and other comorbid psychiatric conditions as potential mediators or moderators, given their substantial role in suicidal ideation and behaviors among individuals with ASD and ADHD traits (63). Clarifying both common and distinct pathways through which these neurodevelopmental traits contribute to suicide risk will be crucial in developing tailored and condition-specific preventive interventions. The scope of study should be expanded to include diverse cultural contexts and populations to enhance generalizability and explore cross-cultural differences in neurodivergent traits and suicide risk. Additionally, the differential effects of specific types of PCEs and ACEs should be more closely examined to better understand their impacts on future mental health outcomes. Finally, researchers must explore additional protective factors, including social support and access to care, to develop more comprehensive intervention strategies for these vulnerable groups. By addressing these gaps, future research can further clarify the complex interplay between neurodivergence, protective factors, and suicide-related behaviors, ultimately informing targeted, effective prevention.

## 5 Conclusions

This study provides novel insights into the relationships between neurodivergent traits, PCEs, and suicide-related behaviors in Japanese youth. The findings reveal that both ASD and ADHD traits independently contribute to an elevated risk of suicidality, and their co-occurrence exacerbates this risk. However, PCEs serve as a significant protective factor against this risk, demonstrating robust effects in mitigating suicide-related behaviors across all levels of ASD and ADHD traits, with particularly pronounced benefits for individuals with higher ADHD trait levels. The results underscore the complex interplay between suicide risk and positive experiences during childhood and suggest that interventions targeting suicide prevention must consider the nuanced roles of neurodivergent traits. In particular, it is critical to address the unique social, emotional, and behavioral challenges experienced by individuals who exhibit both ASD and ADHD traits, as this subgroup demonstrates the highest vulnerability to suicidality. Moreover, fostering PCEs through supportive relationships and nurturing environments during early development may effectively reduce suicide risk in these populations.

## Data Availability

The data analyzed in this study are subject to the following licenses/restrictions: the data are not publicly available because they contain information that could compromise the privacy of research participants. Requests to access these datasets should be directed to Masaki Adachi, adachi@psy.meijigakuin.ac.jp.
