# Supplementary material for: Positive childhood experiences reduce suicide risk in Japanese youth with ASD and ADHD traits: a population-based study
Source: Front Psychiatry. 2025 Apr 30;16:1566098. doi: 10.3389/fpsyt.2025.1566098 (PMC12074681; doi:10.3389/fpsyt.2025.1566098)
Supplement: Supplementary file 1 [file SupplementaryFile1.docx]

Supplementary Material

# Supplementary Figures and Tables

## Supplementary Tables

**Supplementary Table 1.** Sampling by Prefecture

| Number | Prefecture | Sample size (n) | Percentage of total sample size (%) | Estimated population (people) | Percentage of total estimated population (%) |
| --- | --- | --- | --- | --- | --- |
| 1 | Hokkaido | 247 | 4.9 | 5,092,000 | 4.1% |
| 2 | Aomori | 59 | 1.2 | 1,184,000 | 1.0% |
| 3 | Iwate | 61 | 1.2 | 1,163,000 | 0.9% |
| 4 | Miyagi | 127 | 2.5 | 2,264,000 | 1.8% |
| 5 | Akita | 41 | 0.8 | 914,000 | 0.7% |
| 6 | Yamagata | 64 | 1.3 | 1,026,000 | 0.8% |
| 7 | Fukushima | 67 | 1.3 | 1,767,000 | 1.4% |
| 8 | Ibaraki | 88 | 1.8 | 2,825,000 | 2.3% |
| 9 | Tochigi | 65 | 1.3 | 1,897,000 | 1.5% |
| 10 | Gunma | 57 | 1.1 | 1,902,000 | 1.5% |
| 11 | Saitama | 301 | 6.0 | 7,331,000 | 5.9% |
| 12 | Chiba | 274 | 5.5 | 6,257,000 | 5.0% |
| 13 | Tokyo | 589 | 11.8 | 14,086,000 | 11.3% |
| 14 | Kanagawa | 359 | 7.2 | 9,229,000 | 7.4% |
| 15 | Niigata | 82 | 1.6 | 2,126,000 | 1.7% |
| 16 | Toyama | 48 | 1.0 | 1,007,000 | 0.8% |
| 17 | Ishikawa | 48 | 1.0 | 1,109,000 | 0.9% |
| 18 | Fukui | 7 | 0.1 | 744,000 | 0.6% |
| 19 | Yamanashi | 24 | 0.5 | 796,000 | 0.6% |
| 20 | Nagano | 74 | 1.5 | 2,004,000 | 1.6% |
| 21 | Gifu | 69 | 1.4 | 1,931,000 | 1.6% |
| 22 | Shizuoka | 100 | 2.0 | 3,555,000 | 2.9% |
| 23 | Aichi | 327 | 6.5 | 7,477,000 | 6.0% |
| 24 | Mie | 66 | 1.3 | 1,727,000 | 1.4% |
| 25 | Shiga | 56 | 1.1 | 1,407,000 | 1.1% |
| 26 | Kyoto | 144 | 2.9 | 2,535,000 | 2.0% |
| 27 | Osaka | 347 | 6.9 | 8,763,000 | 7.0% |
| 28 | Hyogo | 196 | 3.9 | 5,370,000 | 4.3% |
| 29 | Nara | 57 | 1.1 | 1,296,000 | 1.0% |
| 30 | Wakayama | 16 | 0.3 | 892,000 | 0.7% |
| 31 | Tottori | 17 | 0.3 | 537,000 | 0.4% |
| 32 | Shimane | 32 | 0.6 | 650,000 | 0.5% |
| 33 | Okayama | 76 | 1.5 | 1,847,000 | 1.5% |
| 34 | Hiroshima | 114 | 2.3 | 2,738,000 | 2.2% |
| 35 | Yamaguchi | 39 | 0.8 | 1,298,000 | 1.0% |
| 36 | Tokushima | 23 | 0.5 | 695,000 | 0.6% |
| 37 | Kagawa | 40 | 0.8 | 926,000 | 0.7% |
| 38 | Ehime | 31 | 0.6 | 1,291,000 | 1.0% |
| 39 | Kochi | 25 | 0.5 | 666,000 | 0.5% |
| 40 | Fukuoka | 223 | 4.5 | 5,103,000 | 4.1% |
| 41 | Saga | 30 | 0.6 | 795,000 | 0.6% |
| 42 | Nagasaki | 39 | 0.8 | 1,267,000 | 1.0% |
| 43 | Kumamoto | 62 | 1.2 | 1,709,000 | 1.4% |
| 44 | Oita | 37 | 0.7 | 1,096,000 | 0.9% |
| 45 | Miyazaki | 34 | 0.7 | 1,042,000 | 0.8% |
| 46 | Kagoshima | 65 | 1.3 | 1,549,000 | 1.2% |
| 47 | Okinawa | 53 | 1.1 | 1,468,000 | 1.2% |
| Total | | 5,000 | 100.0 | 124,353,000 | 100.0% |

**Supplementary Table 2.** Post Hoc Multiple Comparisons Using the Holm Method

| **Positive Childhood Experiences** | Difference | Standard error | 95% CI lower class | 95% CI upper class | *t*-value | df | *p*-value | Adjusted  *p*-value | Effect size *d* |
| --- | --- | --- | --- | --- | --- | --- | --- | --- | --- |
| non-sASD+ADHD vs sASD | 0.933 | 0.109 | 0.72 | 1.147 | 8.565 | 4993 | < 0.001 | < 0.001 | 0.416 |
| non-sASD+ADHD vs sADHD | 0.554 | 0.1 | 0.357 | 0.751 | 5.508 | 4993 | < 0.001 | < 0.001 | 0.247 |
| non-sASD+ADHD vs  sASD+ADHD | 1.524 | 0.134 | 1.262 | 1.786 | 11.397 | 4993 | < 0.001 | < 0.001 | 0.68 |
| sASD vs sADHD | −0.38 | 0.138 | −0.651 | −0.108 | −2.741 | 4993 | 0.006 | 0.006 | −0.169 |
| sASD vs sASD+ADHD | 0.591 | 0.164 | 0.27 | 0.912 | 3.605 | 4993 | < 0.001 | 0.001 | 0.263 |
| sADHD vs sASD+ADHD | 0.97 | 0.159 | 0.659 | 1.281 | 6.12 | 4993 | < 0.001 | < 0.001 | 0.433 |
|  |  |  |  |  |  |  |  |  |  |
| **Suicidal Ideation Scale** | Difference | Standard error | 95% CI lower class | 95% CI upper class | *t*-value | df | *p*-value | Adjusted  *p*-value | Effect size *d* |
| non-sASD+ADHD vs sASD | −1.161 | 0.139 | −1.433 | −0.889 | −8.371 | 4993 | < 0.001 | < 0.001 | −0.407 |
| non-sASD+ADHD vs sADHD | −1.436 | 0.128 | −1.687 | −1.185 | −11.218 | 4993 | < 0.001 | < 0.001 | −0.503 |
| non-sASD+ADHD vs sASD+ADHD | −2.587 | 0.17 | −2.921 | −2.253 | −15.192 | 4993 | < 0.001 | < 0.001 | −0.906 |
| sASD vs sADHD | −0.274 | 0.176 | −0.62 | 0.071 | −1.556 | 4993 | 0.12 | *ns* | −0.096 |
| sASD vs sASD+ADHD | −1.425 | 0.209 | −1.835 | −1.016 | −6.830 | 4993 | < 0.001 | < 0.001 | −0.499 |
| sADHD vs sASD+ADHD | −1.151 | 0.202 | −1.547 | −0.755 | −5.7 | 4993 | < 0.001 | < 0.001 | −0.403 |
|  |  |  |  |  |  |  |  |  |  |
| **Suicidal Ideation (Lifetime)** | Difference | Standard error | 95% CI lower class | 95% CI upper class | *t*-value | df | *p*-value | Adjusted  *p*-value | Effect size *d* |
| non-sASD+ADHD vs sASD | −0.083 | 0.022 | −0.127 | −0.039 | −3.708 | 4993 | < 0.001 | < 0.001 | −0.18 |
| non-sASD+ADHD vs sADHD | −0.121 | 0.021 | −0.161 | −0.080 | −5.816 | 4993 | < 0.001 | < 0.001 | −0.261 |
| non-sASD+ADHD vs sASD+ADHD | −0.25 | 0.028 | −0.304 | −0.196 | −9.067 | 4993 | < 0.001 | < 0.001 | −0.541 |
| sASD vs sADHD | −0.037 | 0.029 | −0.093 | 0.019 | −1.304 | 4993 | 0.192 | *ns* | −0.08 |
| sASD vs sASD+ADHD | −0.167 | 0.034 | −0.233 | −0.1 | −4.933 | 4993 | < 0.001 | < 0.001 | −0.36 |
| sADHD vs sASD+ADHD | −0.129 | 0.033 | −0.194 | −0.065 | −3.96 | 4993 | < 0.001 | < 0.001 | −0.28 |
|  |  |  |  |  |  |  |  |  |  |
| **Suicidal Ideation (Past Month)** | Difference | Standard error | 95% CI lower class | 95% CI upper class | *t*-value | df | *p*-value | Adjusted  *p*-value | Effect size *d* |
| non-sASD+ADHD vs sASD | −0.047 | 0.014 | −0.074 | −0.02 | −3.465 | 4993 | 0.001 | 0.002 | −0.168 |
| non-sASD+ADHD vs sADHD | −0.097 | 0.013 | −0.122 | −0.073 | −7.771 | 4993 | < 0.001 | < 0.001 | −0.348 |
| non-sASD+ADHD vs sASD+ADHD | −0.145 | 0.017 | −0.178 | −0.112 | −8.707 | 4993 | < 0.001 | < 0.001 | −0.519 |
| sASD vs sADHD | −0.05 | 0.017 | −0.084 | −0.016 | −2.914 | 4993 | 0.004 | 0.007 | −0.18 |
| sASD vs sASD+ADHD | −0.098 | 0.02 | −0.138 | −0.058 | −4.801 | 4993 | < 0.001 | < 0.001 | −0.351 |
| sADHD vs sASD+ADHD | −0.048 | 0.02 | −0.086 | −0.009 | −2.417 | 4993 | 0.016 | 0.016 | −0.171 |

**Note.** ASD = Autism Spectrum Disorder; ADHD = Attention-Deficit/Hyperactivity Disorder; sASD = suspected ASD; sADHD = suspected ADHD; CI = Confidence Interval; df = degrees of freedom; ns = not significant.


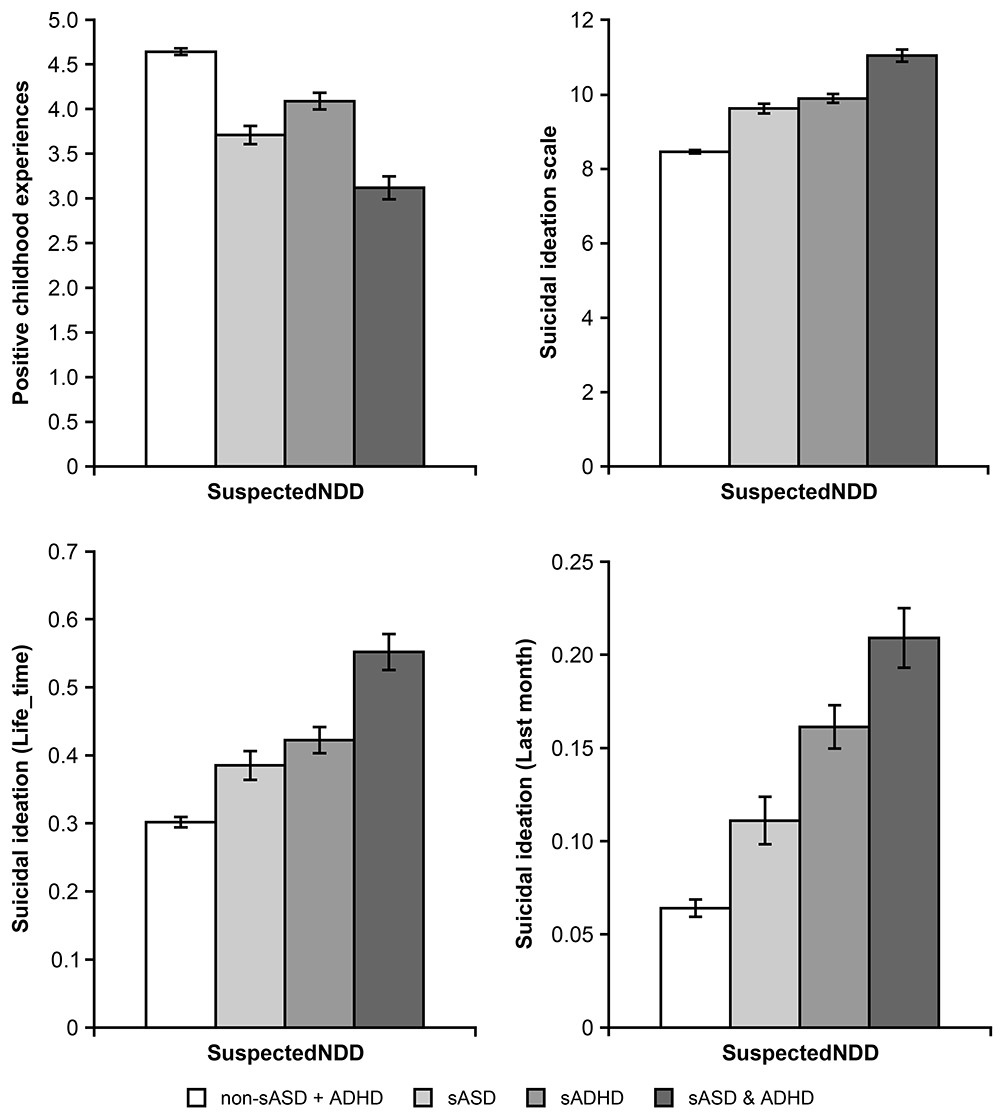


**Suicidal ideation scale**

**Positive childhood experiences**

**Suicidal ideation (past month)**

**Suicidal ideation (Lifetime)**

**Supplementary Figure 1.** Group Differences in Positive Childhood Experiences and Suicide-related Behaviors by Suspected Neurodevelopmental Traits

This figure illustrates group differences across four metrics: PCEs, the Suicidal Ideation Scale, lifetime suicidal ideation, and past-month suicidal ideation. Error bars represent standard errors. The groups include non-sASD+ADHD, sASD, sADHD, and sASD+ADHD. The top left panel shows the PCE scores, in which the non-sASD+ADHD group reported the highest levels of PCEs, whereas the sASD+ADHD group reported the lowest. The top right panel displays the Suicidal Ideation Scale scores, indicating that the sASD+ADHD group exhibited the highest suicidal ideation scores, with progressively lower scores observed across the other groups. The bottom left panel presents lifetime suicidal ideation, with a similar trend of the highest scores being found in the sASD+ADHD group. The bottom right panel shows recent suicidal ideation from the past month, in which the sASD+ADHD group again demonstrated the highest scores.

**Note.** PCEs = Positive Childhood Experiences; ASD = Autism Spectrum Disorder; ADHD = Attention-Deficit/Hyperactivity Disorder; sASD = suspected ASD; sADHD = suspected ADHD.
